# Supplementary figures and images for: A new perspective on the taxonomy and systematics of Arvicolinae (Gray, 1821) and a new time-calibrated phylogeny for the clade
Source: PeerJ. 2024 Jan 9;12:e16693. doi: 10.7717/peerj.16693 (PMC10785794; doi:10.7717/peerj.16693)

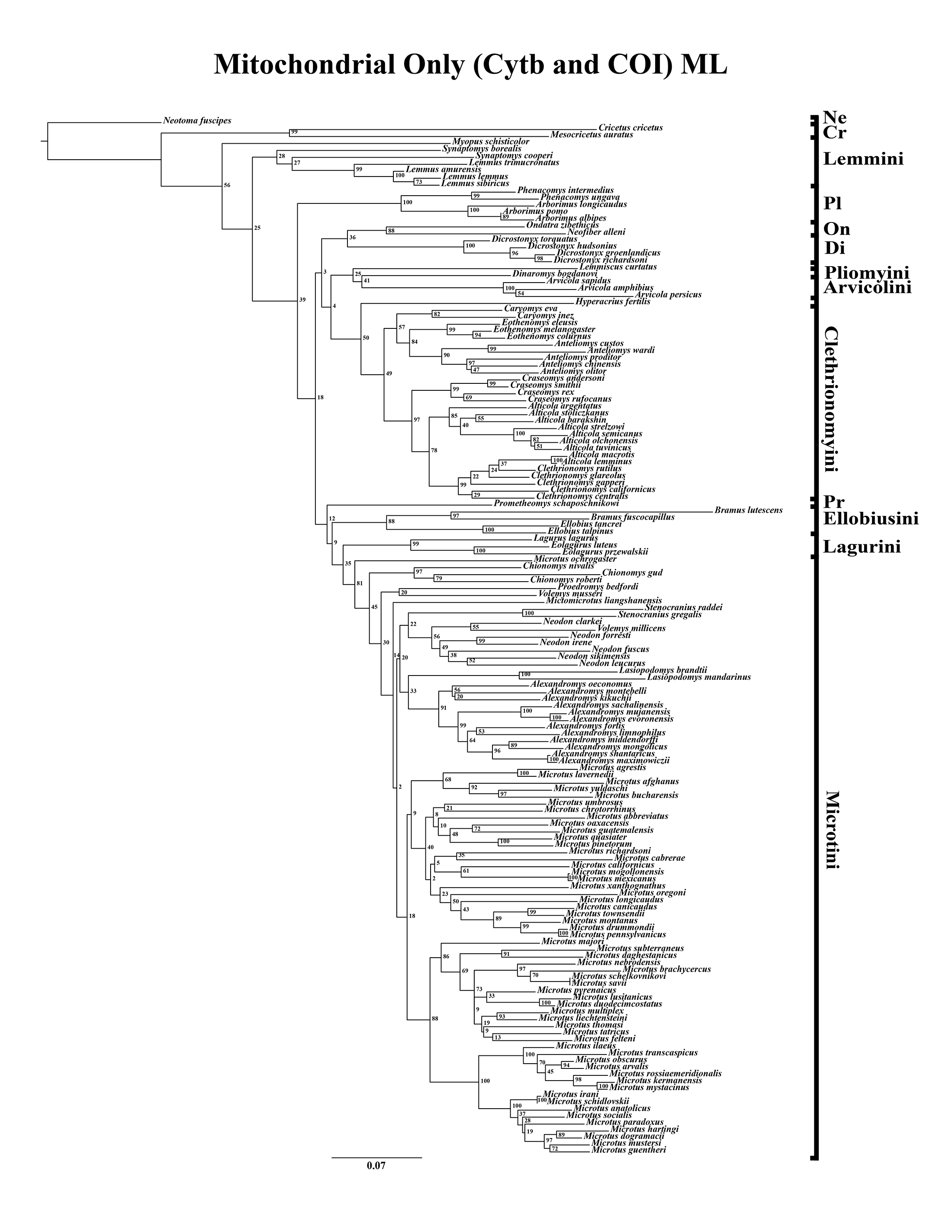

Supplement: Supplemental Information 4 — Tree was rooted with Neotoma fuscipes. Abbreviations: Ne =Neotominae. Cr =Cricetinae. Pl =Pliophenacomys. On =Ondatrini. Di =Dicrostonyxchini. Pr =Prometheomyini. [file peerj-12-16693-s004.png]

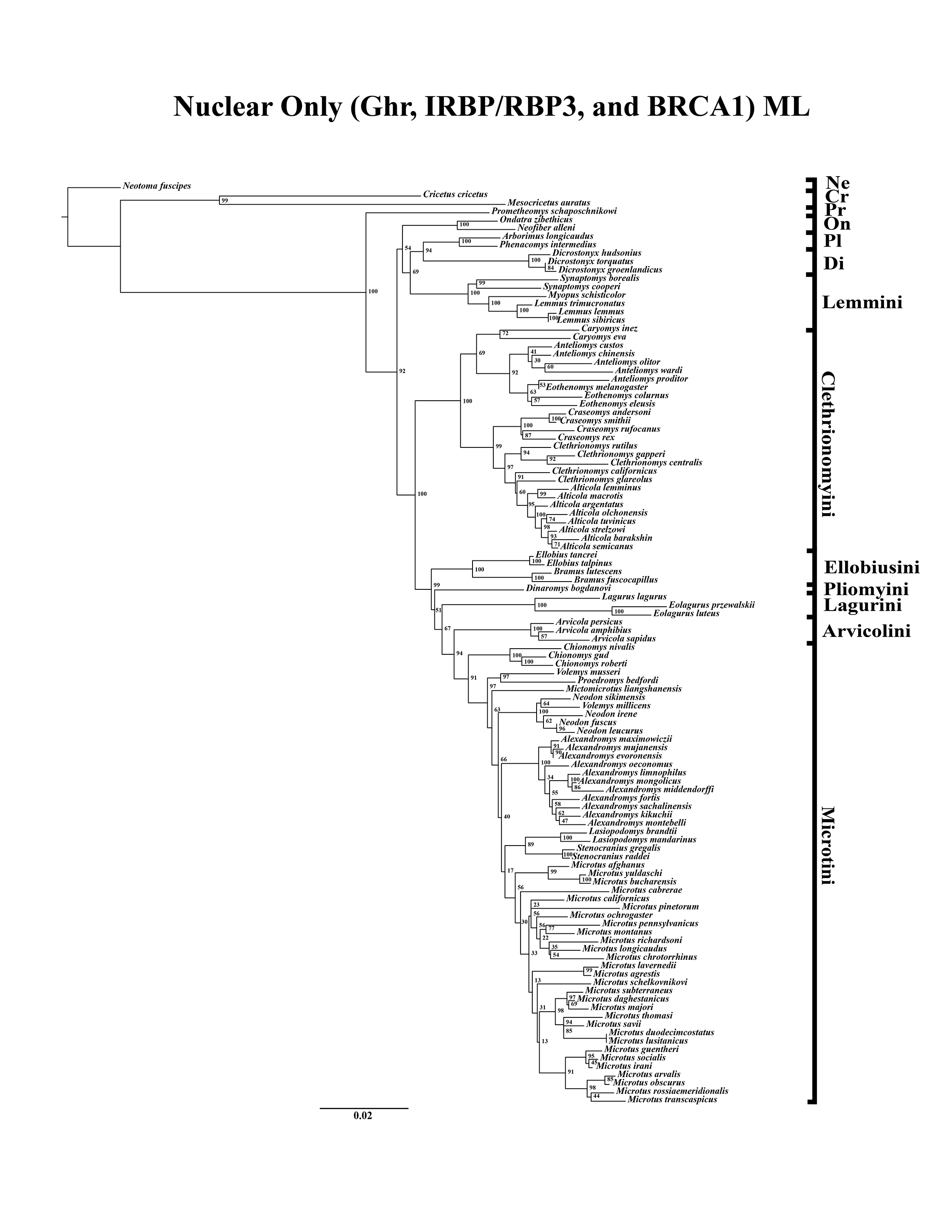

Supplement: Supplemental Information 5 — Tree was rooted with Neotoma fuscipes. Abbreviations: Ne =Neotominae. Cr =Cricetinae. Pl =Pliophenacomys. On =Ondatrini. Di =Dicrostonyxchini. Pr =Prometheomyini. [file peerj-12-16693-s005.png]

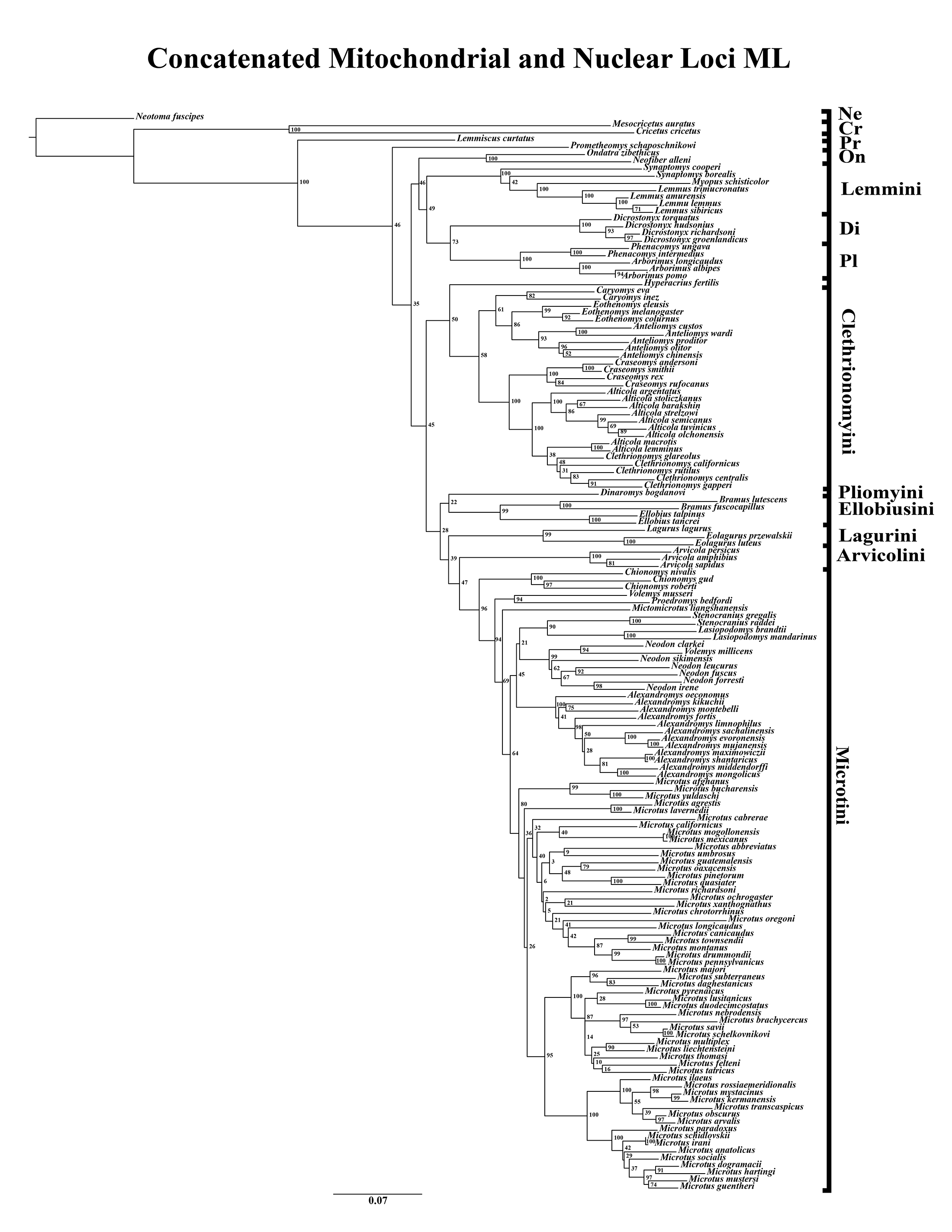

Supplement: Supplemental Information 6 — Tree was rooted with Neotoma fuscipes. Abbreviations: Ne =Neotominae. Cr =Cricetinae. Pl =Pliophenacomys. On =Ondatrini. Di =Dicrostonyxchini. Pr =Prometheomyini. [file peerj-12-16693-s006.png]

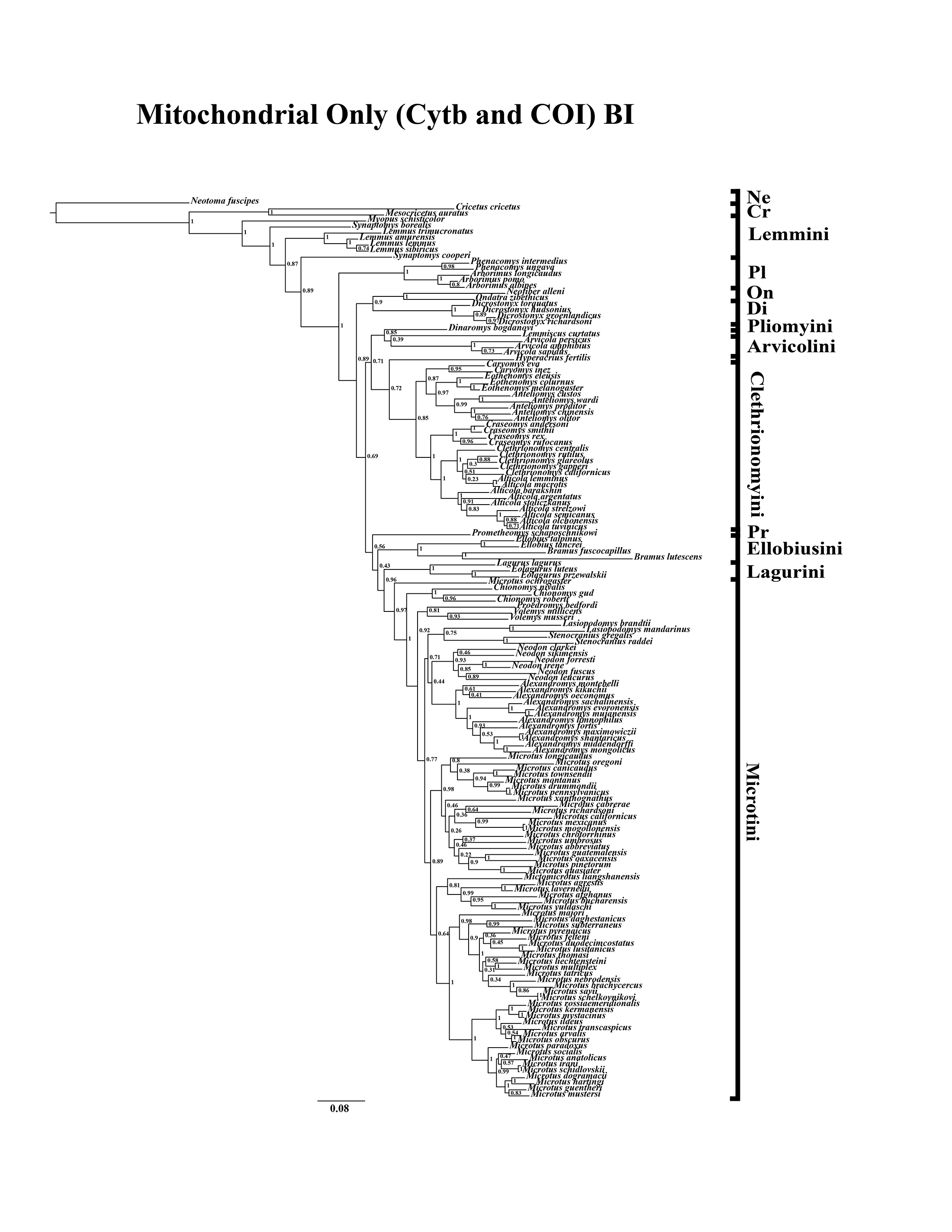

Supplement: Supplemental Information 7 — Abbreviations: Ne =Neotominae. Cr =Cricetinae. Pl =Pliophenacomys. On =Ondatrini. Di =Dicrostonyxchini. Pr =Prometheomyini. [file peerj-12-16693-s007.png]

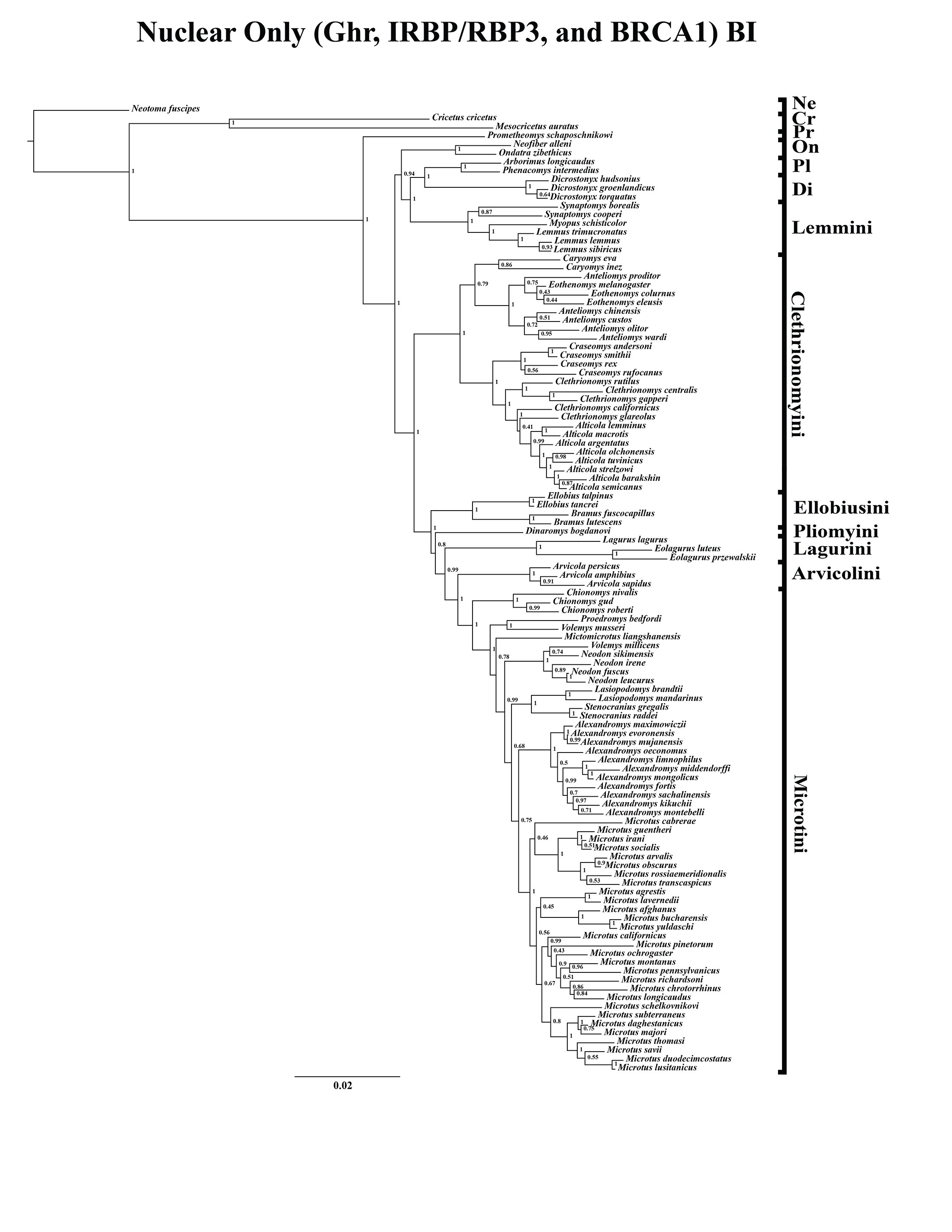

Supplement: Supplemental Information 8 — Abbreviations: Ne =Neotominae. Cr =Cricetinae. Pl =Pliophenacomys. On =Ondatrini. Di =Dicrostonyxchini. Pr =Prometheomyini. [file peerj-12-16693-s008.png]

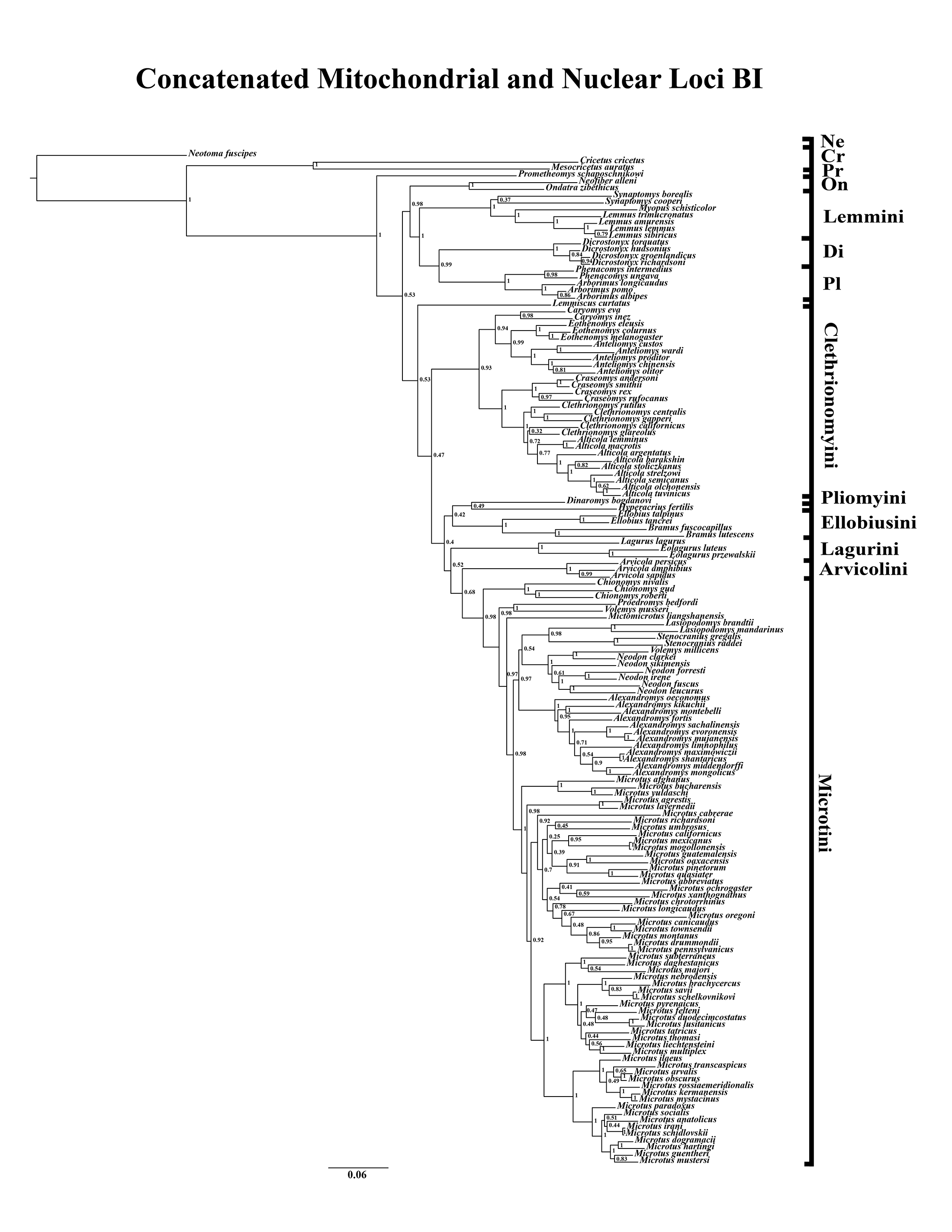

Supplement: Supplemental Information 9 — Abbreviations: Ne =Neotominae. Cr =Cricetinae. Pl =Pliophenacomys. On =Ondatrini. Di =Dicrostonyxchini. Pr =Prometheomyini. [file peerj-12-16693-s009.png]

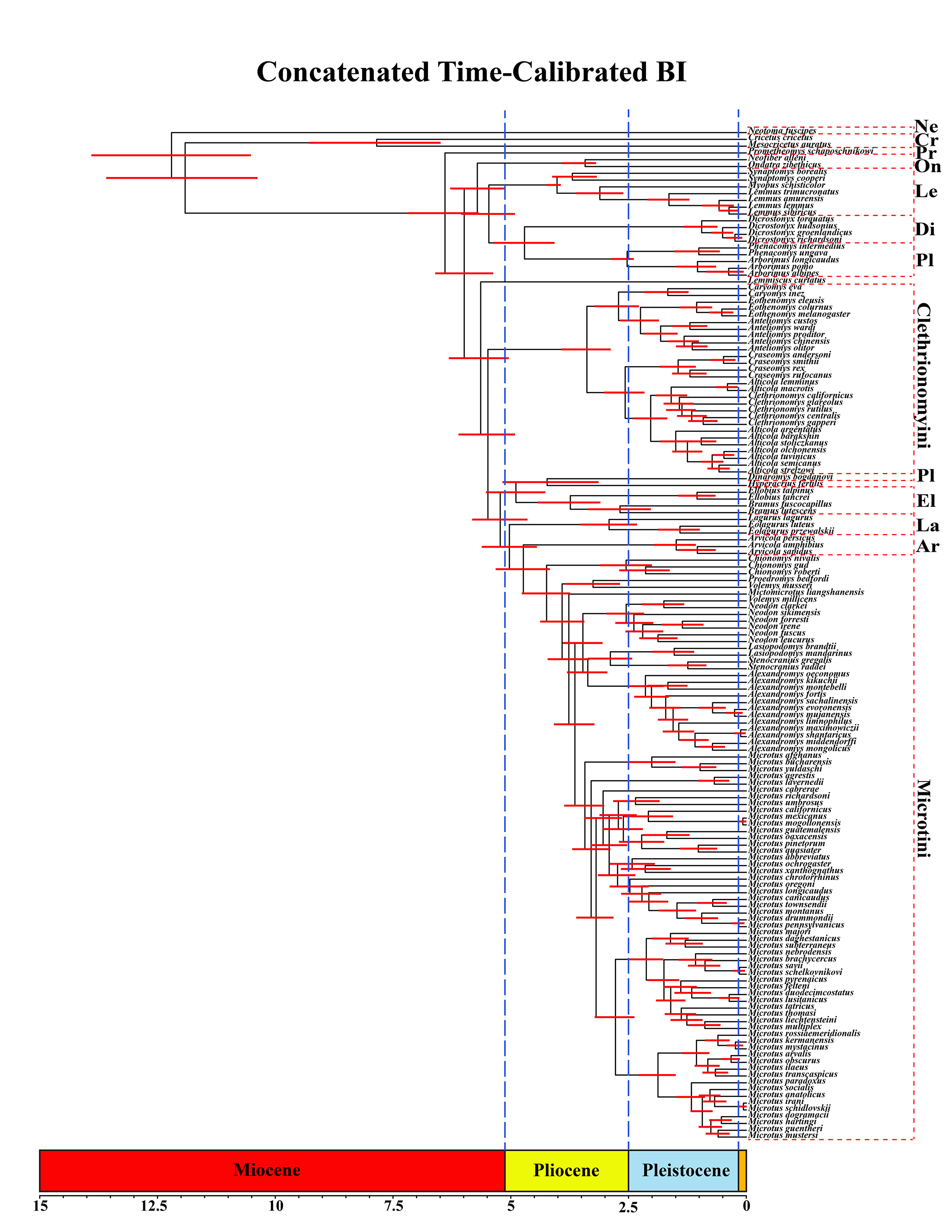

Supplement: Supplemental Information 10 — Blue vertical dashed lines represent boundaries between geologic epoch. Red horizontal bar at each node represent the 95% HPD for the age of the node. Age in scale bar is in millions of years. PLE =Pleistocene. Orange Box =Holocene. [file peerj-12-16693-s010.png]
